# Case Report: Clinicopathological characteristics of patients with gastric cancer with features of a submucosal tumour

**DOI:** 10.3389/fonc.2023.1059815

**Published:** 2023-03-01

**Authors:** Chunnian Wang, Fusang Ye, Huan Zhang, Jie Chen, Lingli Meng, Xianglei He

**Affiliations:** ^1^ Ningbo Clinical Pathology Diagnosis Center, Ningbo, Zhejiang, China; ^2^ Department of Pathology, Zhejiang Provincial People’s Hospital, Hangzhou, Zhejiang, China

**Keywords:** gastric cancer, clinicopathological characteristics, submucosal tumour, pathological diagnosis, differential diagnosis

## Abstract

**Purpose:**

To investigate the clinicopathological characteristics, diagnosis and key points in the differential diagnosis of patients with gastric cancer (GC) with features of a submucosal tumour (GCSMT).

**Methods:**

The clinical presentation and imaging findings of four GCSMT cases diagnosed at our centre from 2016 to 2021 were observed and their clinicopathological outcomes were analysed. The related literature was reviewed. Based on our collected data and the related literature, a total of 31 cases of GCSMT can be summarized.

**Results:**

22 out of 31 cases did not present obvious symptoms and were accidentally discovered during gastroscopic examination. Only 10 patients experienced symptoms such as gastric discomfort, upper abdominal swelling and pain, haematemesis, or haematochezia. The male to female ratio was 22:9 and the age of onset ranged from 40 to 81 years (median age: 63 years). Tumours were located in the upper and middle third of the stomach (24/31), and in the lower third(7/31). The tumour diameter ranged from 0.6 to 7.3 cm, with an average value of 2.5 cm. Endoscopically, the disease manifested as SMTs, with the gastric mucosal surface appearing normal. Most patients underwent radical gastrectomy for GC (80.6%, 25/31). The pathological diagnoses of the 31 cases of GCSMT included well- and moderately-differentiated adenocarcinoma (6/31), poorly differentiated adenocarcinoma or signet ring cell carcinoma 6/31), mucinous adenocarcinoma (9/31), lymphoepithelioma-like carcinoma (7/31), gastric adenocarcinoma of the fundic gland type (3/31). Stage T1b and T2 tumours accounted for 56.7% (17/30) and 26.7% (8/30) of all cases. Lymph node metastases were found in six cases (20.0%, 6/30), whereas distant metastasis was not observed in any of the cases. For the 16 patients whose follow-up data were available, the follow-up time was 5–66 months, during which recurrence or metastasis was not observed.

**Conclusion:**

GCSMT is a rare disease that is often difficult to accurately diagnose through endoscopic biopsy. The importance of gaining an understanding of this disease lies in differentiating it from other SMTs (mostly mesenchymal tumours) to avoid misdiagnosis and missed diagnosis and enable the early diagnosis and treatment of patients.

## 1 Introduction

Gastric cancer (GC) is the fourth most common malignancy worldwide. Endoscopy and pathological biopsy are necessary for its diagnosis. Although the manifestations of this disease vary, the presentation of GC as submucosal tumours (SMTs) is extremely rare. The first case of GC with features of a submucosal tumour (GCSMT) in the English-language literature was reported by Ohara et al. in 1997 (1). Currently, the clinicopathological characteristics of GCSMT remain unclear since a limited number of cases have been reported to date. The diagnosis of GCSMT *via* endoscopic biopsy is also extremely difficult because the tumour cells often remain undetected, even if multiple biopsies are performed. In the present paper, we report four cases of GCSMT and perform a combined review of the data and the related literature to provide an overview of the clinicopathological characteristics and immune phenotypes of GCSMT. We hope that our results can serve as a reference to enhance the knowledge of clinicians and pathologists on this rare tumour type and avoid missed diagnosis and misdiagnosis in clinical practice in the future.

## 2 Materials and methods

### 2.1 Assessment of the new cases

Four cases of GCSMT diagnosed at our centre from 2016 to 2021 were analysed. For all the four cases, the confirmed diagnosis was verified by an experienced and specialised pathologist. The clinical data of patients were reviewed. The pathological specimens were fixed in a 10% neutral-buffered formalin solution, routinely embedded in paraffin and subjected to haematoxylin and eosin staining for morphological characteristic observations. Immunohistochemical staining was performed by En Vision two-step method with diaminobenzidine (DAB) for color development. All antibodies used in this study, including those against CKpan, MUC1, MUC2, MUC5AC, MUC6, Ki67, P53, CD10, CDX2, CEA, CK20, CK7, HER-2, SATB2, EBER, MLH1, MSH2, PMS2 and MSH6, were ready-to-use working solutions purchased from Beijing Zhongshan Biotechnology Co., Ltd. Testing was performed in accordance with the instructions provided by the manufacturer. Positive and negative controls were established for all the immunohistochemically stained samples. Patients were followed-up *via* telephone calls. The cut-off date for follow-up was set as May 10, 2022. This study was approved by the research ethics committee of Ningbo Diagnostic Pathology Center.

### 2.2 Literature review

A review of the literature published in English was performed with MEDLINE search using the terms “gastric cancer with features of a submucosal tumour” or “gastric adenocarcinoma mimicking a submucosal tumor” or “gastric carcinoma resembling submucosal tumor” or “submucosal tumor-like gastric carcinoma”. The references from these articles were also reviewed and the related literature included.

## 3 Results

### 3.1 Clinicopathological characteristics of the new cases

**Figure 1:**
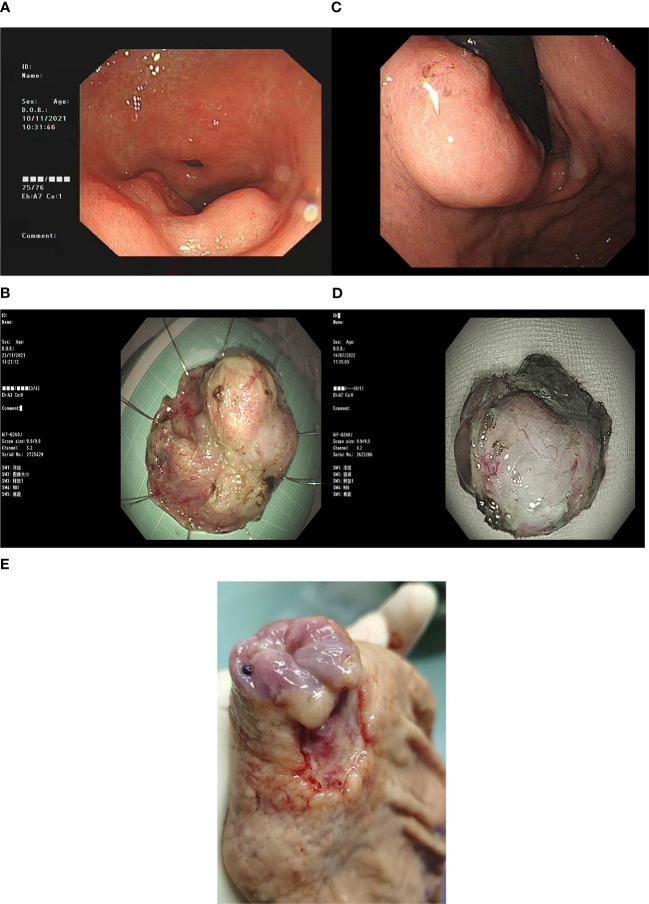
**(A)** ESDGastroscopy of case 1 showed a circumferential uplift lesion on the greater curvature of the antrum, with central depression, the maximum diameter of 5.0cm, and uneven surrounding mucosa. **(B)** ESD surgical resection specimen of Case 1. **(C)** Gastroscopy of Case 3 showed a submucosal bulge below the gastric cardia, with a maximum diameter of 2.0cm, and a shallow ulcer on the surface. **(D)** ESD resection specimen of Case 3. **(E)** In case 3, a gastric cancer radical specimen after ESD showed a mucosal defect in the cardia (caused by).

We reviewed the clinical data and pathological characteristics of the four cases(see **Table 1**). All of the four patients were men and they were aged 58–69 years. The clinical symptoms were not specific. Three cases were discovered during a health check-up (space-occupying lesion in the stomach), and one case manifested as gastric discomfort accompanied by an elevated serum CA19-9 level. The disease manifested endoscopically as SMTs (**Figures 1A. B**), with the lesion size ranging from 1.3 to 3.5 cm. Three lesions were located in the upper third of the stomach and one in the lower third of the stomach. No GC diagnosis was obtained after multiple gastroscopic biopsies. Three patients underwent local mass resection (**Figures 1C, D**), followed by radical gastrectomy for GC (**Figure 1E**), one underwent endoscopic submucosal dissection (ESD).

**Table 1:** Clinical information of 4 GCSMT patients in our group.

No.	Sex	Age	Symptoms	Tumourlacation	Tumoursize	Imaging and endoscopic examination	Clinicaltreatments	pTNMclassification	Type of tissue	Follow-up
1	Male	67	asymptomat ic	L	3.5cm	Gastroscopy showed a circumferential uplift lesion on the greater curvature of the antrum with central depression, the maximum diameter was 5.0cm, and the surrounding mucosa was uneven, and there were no abnormal microvessels andmicrostructures on the surface.	radical resection	pT1bN1M0	mucinous adenocarcinoma	5 months, no recurrence or metastasis
2	Male	58	Epigastric discomfort	L	3.0cm	Gastroscopy showed a submucosal bulge in the prepyloric region	radical resection	pT2N0M0	mucinous adenocarcinoma	15 months, no recurrence ormetastasis
3	Male	69	asymptomat ic	U	2.0cm	Endoscopic ultrasonography showed hypoechoic lesions under the gastric body, suggesting thepossibility of submucosal cyst.	radical resection	pT2N0M0	mucinous adenocarcinoma	34 months, no recurrence ormetastasis
4	Male	59	asymptomat ic	L	1.3cm	Gastroscopy showed submucosal uplift in the anterior wall of the antrum.	ESD	pT1bN0M0	lymphoepithelio ma-likecarcinoma	66 months, no recurrence ormetastasis

L, Lower body of the stomach; M,middle body of the stomach; U, upper body of the stomach; pTNM, pathological tumour-node-metastasis; ESD, endoscopic submucosal dissection.

The local mass resection specimens of the four cases, including the ESD specimens, were dissected along the largest lesion diameter perpendicular to the mucosal surface, to examine the lesions. Tumours were mainly located in the submucosa. The size of the tumours was in the 1.3–3.5 cm range, and they exhibited a greyish-white cut surface and unclear boundaries, and were mucinous in certain cases (75%, 3/4). For the three patients who underwent radical gastrectomy after local mass resection, specimens were collected using the routine procedure for radical gastrectomy specimen extraction, and the residual masses in the specimens were not observed by the naked eye.

**Figure 2:**
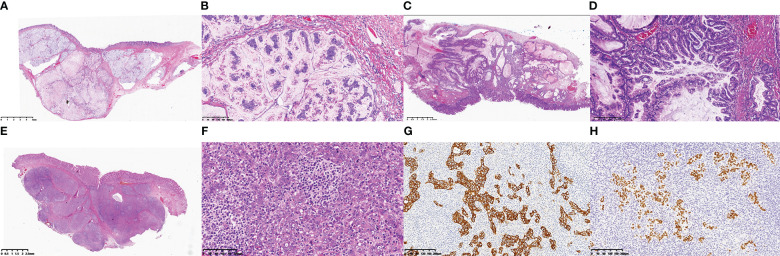
**(A)** Under low power microscope, case 1 showed that the tumor was located in the submucosa and the surface epithelium was intact. The tumor was nodular distribution, rich in mucus with floating cancer cells. **(B)** In case 1, the tumor cells showed dichromatic cytoplasm, large nuclei, prominent nucleoli, arranged in irregular nests, and located in the mucus pool. **(C)** In case 3, the tumor was located in the submucosa, rich in mucus, and locally continuous with the surface epithelium. **(D)** In case 3, the tumor cells were columnar, with abundant cytoplasm and mucus, and small nuclei located at the base. The tumor cells were arranged in glandular and papillary patterns. **(E)** In case 4, the tumor was located in the submucosa with nodular distribution, and the stroma was rich in lymphocytes with lymphoid follicles. **(F)** In case 4, the tumor cells were syncytial, with chromophilic cytoplasm, large nuclei, prominent nucleoli, and arranged in irregular nests. The immunohistochemical results of case 4 showed that the tumor cells expressed CKpan **(G)** and EBER **(H)**.

Three cases of mucinous adenocarcinoma were found, among which two were well-differentiated mucinous adenocarcinomas and one was a poorly differentiated mucinous adenocarcinoma. Another case had gastric carcinoma with lymphoid stroma (GCLS, i.e., lymphoepithelioma-like carcinoma). Under the microscope, the SMT specimen of the patient with a poorly differentiated mucinous adenocarcinoma exhibited a nodular distribution with copious extracellular mucin (**Figure 2A**). Tumour cells were epithelium-like, possessed a large nucleus and distinct nucleoli, and floated within mucin pools in small nest-like clusters (**Figure 2B**). Necrotic nuclear fragments were also observed. The microscopic examination of the well-differentiated mucinous adenocarcinoma specimens revealed the presence of dense hyperplasia of the submucosal glands, a large nucleus, distinct nucleoli, obvious nuclear division, and local glandular fusion. This was locally accompanied by the formation of mucin pools (**Figures 2C, D**). Certain regions exhibited glandular ulcerations with spill over of mucin into the fibrous mesenchyme, which was accompanied by hyperplasia of the fibrous granulation tissue. Histological examination of the SMT specimens of case 4 showed the presence of a nodular structure and a lymphocyte-rich mesenchyme, accompanied by lymphoid follicle formation (**Figure 2E**). At high magnification, the tumour cells exhibited a symplastic appearance with a dichroic cytoplasm, a large nucleus, distinct nucleoli, and an irregular nest- and sheet-like arrangement (**Figure 2F**). The involvement of the muscularis propria was found in certain cases (50%, 2/4). The tumor cells were found to have migrated to the epithelial cells of the mucosal layer in only two cases. Among the three patients who underwent extended resection, one exhibited a small amount of local residual mucinous adenocarcinoma in the submucosa and metastasis in one out of 18 lymph nodes. In the other two cases, no metastasis was found in lymph nodes dissected.

The tumour cells of the four cases showed differing expression levels of the gastric mucins MUC1 (4/4), MUC5AC (2/4), MUC6 (2/4), CEA (4/4), CKpan (4/4)(**Figure 2G**) and CK7 (3/4). The intestinal markers MUC2, CDX2 and CK20 were expressed in case 1, and EBER was expressed in the tumour cells of case 4 (**Figure 2H**). Ki-67 expression indicated that the tumour cell proliferation index was in the 20%–70% range. The tumor cells of four cases did not express CD10 and SATB2 proteins, no mutant expression of P53, and no deletion of mismatch repair proteins MLH1, PMS2, MSH2 and MSH6.

### 3.2 Literature review

As of December 2021, a total of 27 English-language reports of GCSMT with adequate clinicopathological data have been found in the literature (2–24). Therefore, we performed a combined review of the five cases described above and the related literature, as shown in **Table 2**. 22 out of 31 cases did not present obvious symptoms and were accidentally discovered during gastroscopic examination. Only 10 patients experienced symptoms such as gastric discomfort, upper abdominal swelling and pain, haematemesis, or haematochezia. The male to female ratio was 22:9 and the age of onset ranged from 40 to 81 years (median age: 63 years). Tumours were located in the upper and middle third of the stomach (24/31), and in the lower third(7/31). The tumour diameter ranged from 0.6 to 7.3 cm, with an average value of 2.5 cm. Endoscopically, the disease manifested as SMTs, with the gastric mucosal surface appearing normal in most cases and being accompanied by a central indentation in certain cases. Some patients with mucinous adenocarcinoma exhibited umbilicus-like indentations on the tumour mucosal surface, accompanied by mucin exudation.

**Table 2:** Clinicopathological data of 31 patients with GCSMT.

No.	Author	Sex	Age	Symptoms	Tumour lacation	Tunour size	Clinical treatments	pTNMclassification	Type of tissue	Follow-up
1	Ohara (1)	Male	48	Epigastricdiscomfort	L	2.0cm	radical resection	pT3N0M0	well differentiatedadenocarcinoma	18 months, no recurrence ormetastasis
2	Hosoda (2)	Male	71	asymptomatic	M	1.2cm	radical resection	pT1bN2M0	poor differentiatedadenocarcinoma	unknown
3	Umehara (3)	Male	50	hematemesis	U	3.5cm	radical resection	pT2N0M0	poor differentiatedadenocarcinoma	unknown
4	Kume (4)	Female	49	abdominalpain	M	1.0cm	radical resection	pT1bN0M0	well differentiatedadenocarcinoma	unknown
5	Fujiyoshi (5)	Female	73	asymptomatic	U	1.0cm	radical resection	pT1bN0M0	moderately differentiatedadenocarcinoma	18 months metastasis,26 months, unexplained death
6	Takahashi (6)	Male	50	asymptomatic	M	2.0cm	radical resection	pT2N0M0	lymphoepithelioma-likecarcinoma	8 months, no recurrence ormetastasis
7	Teraishi (7)	Male	63	asymptomatic	M	2.5cm	radical resection	pT2N0M0	moderately differentiatedadenocarcinoma	unknown
8	Ando (8)	Female	65	anemia, tarrystools	M	2.0cm	radical resection	pT1bN0M0	mucinous adenocarcinoma	unknown
9	Kim (9)	Male	66	difficultyswallowing	U	7.3cm	radical resection	pT2N0M0	mucinous adenocarcinoma	unknown
10	Kim (10)	Male	46	asymptomatic	U	2.5cm	radical resection	pT1bN0M0	lymphoepithelioma-likecarcinoma	unknown
11	Yu (11)	Female	54	dyspepsia,vomiting	L	3.1cm	local resection	pT3NXMO	mucinous adenocarcinoma	unknown
12	Yu (11)	Female	50	asymptomatic	M	5.0cm	radical resection	pT4aN2M0	mucinous adenocarcinoma	unknown
13	Yoo (12)	Female	40	asymptomatic	U	2.5cm	radical resection	pT1bN0M0	mucinous adenocarcinoma	unknown
14	Matsumoto (13)	Male	58	asymptomatic	U	unknown	local resection	pT1bN0M0	lymphoepithelioma-likecarcinoma	unknown
15	Imamura (14)	Male	66	asymptomatic	U	2.0cm	radical resection	pT1bN0M0	well differentiatedadenocarcinoma	12 months, no recurrence ormetastasis
16	Cha (15)	Male	69	asymptomatic	U	2.4cm	radical resection	unknown	adenocarcinoma of thefundic gland	unknown
17	Chen (16)	Male	50	Epigastricdiscomfort	M	2.2cm	radical resection	pT1bN0M0	lymphoepithelioma-likecarcinoma	12 months, no recurrence ormetastasis
18	Li (17)	Male	44	paroxysmalepigastric pain	U	1.0cm	local resection	pT1bN0M0	poor differentiatedadenocarcinoma	unknown
19	Yamane (25)	Male	81	asymptomatic	M	3.5cm	radical resection	pT2N1M0	poor differentiatedadenocarcinoma	18 months, no recurrence ormetastasis
20	Jin Lee (18)	Female	64	asymptomatic	M	3.5cm	radical resection, postoperativechemotherapy	pT4aN3M0	signet ring cell carcinoma	6 months, no recurrence or metastasis
21	Ozawa (19)	Male	80	asymptomatic	M	2.0cm	radical resection	pT4aN0M0	moderately differentiatedadenocarcinoma	6 months, no recurrence ormetastasis
22	Kobayashi (20)	Female	72	asymptomatic	M	2.0cm	radical resection	pT1bN0M0	lymphoepithelioma-likecarcinoma	14 months, no recurrence ormetastasis
23	Kobayashi (20)	Male	73	asymptomatic	L	1.9cm	ESD	pT1bN0M0	lymphoepithelioma-likecarcinoma	12 months, no recurrence ormetastasis
24	Cheng (21)	Male	50	asymptomatic	L	1.6cm	radical resection	pT1bN2M0	poor differentiatedadenocarcinoma	6 months, no recurrence ormetastasis
25	Uchida (22)	Male	70	asymptomatic	M	2.0cm	radical resection	pT1bN0M0	adenocarcinoma of the	12 months, no recurrence or
									fundic gland	metastasis
26	Yu (23)	Female	77	Epigastricdiscomfort	M	0.6cm	ESD	pT1bN0M0	adenocarcinoma of thefundic gland	unknown
27	Kim (24)	Male	74	asymptomatic	M	4.5cm	radical resection	pT2N0M0	mucinous adenocarcinoma	unknown
28	Our case 1	Male	67	asymptomatic	L	3.5cm	radical resection	pT1bN1M0	mucinous adenocarcinoma	5 months, no recurrence ormetastasis
29	Our case 2	Male	58	Epigastricdiscomfort	L	3.0cm	radical resection	pT2N0M0	mucinous adenocarcinoma	15 months, no recurrence ormetastasis
30	Our case 3	Male	69	asymptomatic	U	2.0cm	radical resection	pT2N0M0	mucinous adenocarcinoma	34 months, no recurrence ormetastasis
31	Our case 4	Male	59	asymptomatic	L	1.3cm	ESD	pT1bN0M0	lymphoepithelioma-likecarcinoma	66 months, no recurrence ormetastasis

L, Lower body of the stomach; M,middle body of the stomach; U, upper body of the stomach; pTNM, pathological tumour-node-metastasis; ESD, endoscopic submucosal dissection.

Most patients underwent radical gastrectomy for GC (80.6%, 25/31). Three patients with lymphoepithelioma-like carcinoma or gastric adenocarcinoma of the fundic gland type whose tumours were classified as pT1N0M *via* pTNM staging merely underwent ESD, while the remaining three patients underwent local gastric wall resection (one case of pT3NXMO mucinous adenocarcinoma, one case each of pT1bN0M0 lymphoepithelioma-like carcinoma and poorly differentiated adenocarcinoma). The pathological diagnoses of the 31 cases of GCSMT included well- and moderately-differentiated adenocarcinoma (6/31), poorly differentiated adenocarcinoma or signet ring cell carcinoma (6/31), mucinous adenocarcinoma (9/31), lymphoepithelioma-like carcinoma (7/31), gastric adenocarcinoma of the fundic gland type (3/31). Stage T1b and T2 tumours accounted for 56.7% (17/30) and 26.7% (8/30) of all cases. Lymph node metastases were found in six cases (20.0%, 6/30), whereas distant metastasis was not observed in any of the cases. For the 16 patients whose follow-up data were available, the follow-up time was 5–66 months, during which recurrence or metastasis was not observed. The patients had generally satisfactory outcomes, which may be related to the fact that most patients were followed-up for less than 18 months (68.8%, 11/16).

## 4 Discussion

Gastric SMTs are usually caused by various neoplastic or non-neoplastic diseases in the submucosa or in deeper tissues of the stomach, including gastrointestinal stromal tumours, neurilemmomas, lipomas, and lymphomas. GCSMT refers to a GC that presents endoscopically as an SMT, with the surface mucosa appearing normal or manifesting as local foci accompanied by small umbilicus-like indentations. It is a rare disease that accounts for only 0.1%–0.63% of all GC cases (21, 25). The clinicopathological characteristics of GCSMT remain unclear since only a limited number of cases have been reported to date.

Among the cases of asymptomatic SMTs discovered during routine endoscopy, most were non-epithelial tumours, and only a small number of them were GCSMT. Certain differences in the endoscopic morphology of GCSMTs and submucosal non-epithelial tumours exist. For instance, the former manifest as low-height protrusions, a disordered base region, visible indentations on the surface, which occupy a large area of the entire lesion, and shallow depressions on the surface.

Mucinous adenocarcinoma is a histological subtype of adenocarcinoma. In GC, gastric mucinous adenocarcinoma is relatively rare and accounts for only 2.1%–8.1% of all GC cases (26). However, up to 29.0% of the 31 GCSMT cases reviewed in this study were mucinous adenocarcinoma cases (9/31). Early mucinous adenocarcinoma is even rarer, with most cases of early gastric mucinous adenocarcinoma manifesting as SMTs. Therefore, when SMTs are found endoscopically, the possibility of an early mucinous adenocarcinoma diagnosis should be considered. During endoscopy, attention should be paid to subtle changes, such as the presence or absence of erosion, depressions, and the state of the tumour margins. When necessary, endoscopic ultrasonography-guided fine needle aspiration biopsy or SMT resection may be performed to confirm the diagnosis at an early stage and avoid treatment delay as much as possible. Some researchers believe that mucinous adenocarcinoma arises initially as a typical adenocarcinoma, and then becomes mucinous with tumour progression. This is consistent with the decreased intraluminal mucoprotein excretion and increased intramural mucoprotein accumulation observed following the tumour infiltration of the gastric wall (27). We deduce that mucin accumulation within the gastric wall may be the main cause of the SMT-like appearance of GCSMTs. The biological behaviour of gastric mucinous adenocarcinoma is similar to that of non-mucinous GC, and its outcomes are related to the tumour differentiation, size and location, the depth of infiltration, lymph node metastasis and clinical staging, and not to the mucoprotein content. The multivariate analysis results indicated that the mucinous histological subtype is not an independent prognostic factor of patients with GC; however, patients with early gastric mucinous adenocarcinoma have good postoperative outcomes (4). Among the nine cases of mucinous adenocarcinoma reviewed in the present paper, eight were treated *via* radical gastrectomy for GC and one was treated *via* local gastric wall resection. The three patients whose follow-up data were available (one patient with pT1bN0M0 disease and two patients with T2N0M0 disease), were followed-up for 5–34 months, during which no recurrence or metastasis was observed.

Lymphoepithelioma-like carcinoma, also known as gastric carcinoma (adenocarcinoma) with lymphoid stroma (GCLS), accounts for 1.0%–7.0% of all GC cases (26) and approximately 22.6% of the 31 GCSMT cases reviewed in this paper (7/31). Kobayashi et al. analysed 11 cases of lymphoepithelioma-like carcinoma, among which three cases (27.3%, 3/11) endoscopically exhibited an SMT-like morphology. Results indicated that the disease was associated with the male sex, a proximal location, and an SMT-like appearance. Diagnosis was difficult since five cases (45.5%) were negative upon pathological examination *via* biopsy. The tumour invasion depth was more than 1,000 µm into the submucosa in all cases. In the five patients who underwent radical gastrectomy for GC, no lymph node metastasis was observed. Follow-up was performed for 12–48 months, and neither local recurrence nor distant metastasis was reported during the follow-up period. The researchers asserted that submucosa-invasive lymphoepithelioma-like carcinoma can be entirely excised endoscopically to avoid excessive and unnecessary gastrectomy (20). All seven cases of lymphoepithelioma-like carcinoma reviewed in this paper were treated using gastrectomy or ESD, among which six had stage T1b disease and one had stage T2 disease. Postoperative pathological examinations showed an absence of lymph node metastasis. The patients were followed-up for 8–66 months, during which recurrence or metastasis was not observed. More data are needed to further study the indications for ESD treatment and the prognosis of patients with lymphoepitheliomatoid carcinoma.

Several pathological mechanisms related to the occurrence of GCSMT have been described in the literature, including lymphocyte infiltration in lymphoepithelioma-like carcinoma, mucin aggregation in mucinous adenocarcinoma, adenocarcinoma arising from a heterotopic pancreas in the gastric wall, GC arising from gastritis cystica profunda (GCP) (6) and excessive fibrosis in the surrounding tissues of GC (28).

Differential diagnoses may be performed in the following cases: 1. GCP: clinical and endoscopic observations may be similar; however, a history of gastric surgery is usually present in GCP cases. In the absence of surgical history, multiple cysts may be observed in the hypertrophic submucosal layer *via* endoscopic ultrasonography. The presence of non-atypic, well-differentiated dilated glands of various sizes within and below the muscularis mucosae can be observed by histological examination; 2. Heterotopic pancreas: there is usually a lack of clinical manifestations of a heterotopic pancreas. The condition is usually accidentally discovered during surgery or examination. In approximately 90% of cases, the heterotopic pancreas is located in the upper gastrointestinal tract, mainly in the stomach (usually on the side of the greater curvature within 5 cm from the pylorus), duodenum and jejunum. It usually manifests endoscopically as submucosal protrusion lesions, accompanied by a central umbilicus-like indentation. The pancreatic ducts, acini and/or islets can be observed during pathological examination, along with non-atypic cells. 3. Metastasis of pancreatic cancer in the gastric wall induced by endoscopic ultrasound-guided fine needle aspiration (29): a differential diagnosis is mainly made based on the medical history and lesion site. 4. Metastatic cancer: a differential diagnosis can be made based on the medical history and the morphological and immunohistochemical indicators of the actual tumour.

Endoscopic biopsy of GCSMT usually produces a negative result, increasing the tendency of the occurrence of missed diagnosis or misdiagnosis as non-epithelial tumours, thereby resulting in delayed treatment. Upon the observation of high-risk characteristics, such as irregular boundaries, surface ulcerations, an obvious volume increase, intra-lesion heterogeneity or local lymphadenectasis, biopsy or endoscopic local resection should be performed in a timely manner to avoid treatment delay. A larger number of cases are required for further investigation to enhance our understanding of the disease and guide future clinical practice.

## Data availability statement

The original contributions presented in the study are included in the article/supplementary material. Further inquiries can be directed to the corresponding author.

## Ethics statement

Written informed consent was obtained from the individual(s) for the publication of any potentially identifiable images or data included in this article.

## Author contributions

Analysis of the results and the writing of the manuscript: CW. Collection of data: FY, HZ, JC and LM. Analysis of the results and pathological pictures: CW, FY and HZ. Design and implementation of the research and revision of the manuscript: XH. All authors contributed to the article and approved the submitted version.
